# Adhesion-independent synergy of monocytes and endothelial cells in cytokine production: regulation of IL-6 and GM–CSF production by PAF

**DOI:** 10.1155/S0962935196000105

**Published:** 1996-02

**Authors:** C. Lacasse, S. Turcotte, D. Gingras, M. Rola-Pleszczynski

**Affiliations:** 1 Immunology Division Department of Pediatrics Faculty of Medicine Université de Sherbrooke Sherbrooke QC Canada; 2 Phoenix International Life Sciences Inc. 4625 Dobrin St. St-Laurent QC Canada

## Abstract

Co-Cultures of monocytes (MO) and endothelial cells (EC) were studied for their capacity to synergize in the production of interleukin-6 (IL-6) and granulocyte-macrophage colony-stimulating factor (GM–CSF), two cytokines potentially important in vascular physiopathology. Resting monocytes produced detectable amounts of IL-6 but no GM–CSF, whereas confluent EC produced significant quantities of GM–CSF, but minimal IL-6. In co-cultures without stimuli, additive synthesis of both cytokines was observed. When EC were pretreated, however, with either PAF, TNF or both stimuli, before addition of MO, synergistic production of IL-6 was observed. In contrast, GM–CSF production was not enhanced by coculture of monocytes with activated EC. When either cell population was fixed with paraformaldehyde or killed by freeze-thawing before addition to the co-culture, cytokine levels reverted to those produced by the unaffected population alone. On the other hand, separating the two cell populations by a cell-impermeable membrane in transwell cultures did not affect the synergistic production of the cytokines. Taken together, our data suggest that EC and MO can synergize in response to stimuli by producing IL-6 and that this synergy is dependent on the integrity of both cell populations, but independent of cell-cell contact.


Mediators of Inflammation 5, 56–61 (1996)

### Adhesion-independent synergy of monocytes and endothelial cells in cytokine production: regulation of IL-6 and GM–CSF production by PAF

C. Lacasse,* S. Turcotte, D. Gingras and M. Rola-Pleszczynski[CA]

Immunology Division, Department of Pediatrics, Faculty of Medicine, Université de Sherbrooke, Sherbrooke, QC, Canada. Fax: (+1) 819 8206414.
*Present address: Phoenix International Life Sciences Inc., 4625 Dobrin St., St-Laurent, QC, Canada

[CA]Corresponding Author

**Co-cultures of monocytes (MO) and endothelial cells (EC) were studied for their capacity to synergize in the production of interleukin-6 (IL-6) and granulocyte-macrophage colony-stimulating factor (GM–CSF), two cytokines potentially important in vascular physiopathology. Resting monocytes produced detectable amounts of IL-6 but no GM–CSF, whereas confluent EC produced significant quantities of GM–CSF, but minimal IL-6. In co-cultures without stimuli, additive synthesis of both cytokines was observed. When EC were pretreated, however, with either PAF, TNF or both stimuli, before addition of MO, synergistic production of IL-6 was observed. In contrast, GM–CSF production was not enhanced by co-culture of monocytes with activated EC. When either cell population was fixed with paraformaldehyde or killed by freeze–thawing before addition to the co-culture, cytokine levels reverted to those produced by the unaffected population alone. On the other hand, separating the two cell populations by a cell-impermeable membrane in transwell cultures did not affect the synergistic production of the cytokines. Taken together, our data suggest that EC and MO can synergize in response to stimuli by producing IL-6 and that this synergy is dependent on the integrity of both cell populations, but independent of cell–cell contact.**

**Key words:** Endothelial cells, GM–CSF, IL-6, Monocytes, PAF, Synergy

### Introduction

Vascular endothelium interacts closely with leukocytes and constitutes an important cellular element in inflammatory and immunologic responses.[1] This interaction is in part regulated by cytokines and several other mediators derived from leukocytes which act on endothelial cells (EC).[2–4] Among these, platelet-activating factor (PAF) can be produced by a variety of cells such as monocytes, neutrophils and endothelial cells.[5–8] This phospholipid is a potent pro-inflammatory mediator and causes a variety of effects, including microvascular leakage, contraction of smooth muscle and activation of neutrophils, macrophages and eosinophils.[9–11] We have demonstrated that PAF can induce suppressor cell activities in mononuclear leukocytes *in vitro*,[12,13] as well as stimulate IL-1, TNF and IL-6 production in monocytes and alveolar macrophages.[14,15] In cultured human EC, PAF was shown to induce shape changes, release of prostacyclin and vasodilatation.[16,17] PAF is also proposed as a key player in leukocyte adhesion to the endothelium by tethering leukocytes and inducing subsequent activation and adhesion stages.[18]

Interleukin-6 (IL-6) has been shown to be a multifunctional cytokine that regulates, among other things, immune responses, acute phase reactions and haematopoiesis. It has also been reported to induce EC proliferation.[19] IL-6 is synthesized by EC following stimulation with IL-1, LPS or TNF-α,[20–22] but other cells also produce IL-6, including monocytes, macrophages and fibroblasts.[23–26]

EC can also synthesize GM–CSF, either constitutively or more commonly after stimulation with IL-1[27–31] or TNF.[32] The production of GM–CSF by fibroblasts,[33] T lymphocytes,[34] mast cells[35] and monocytes/macrophages[36] has also been demonstrated. GM–CSF is an important glycoprotein in the interaction of leukocytes with EC since it promotes the adherence of monocytes to EC,[37] augments accessory cell function of monocytes and stimulates certain monocyte effector activities involved in microbial and tumoricidal killing.[38] In the present work, we examined the potential synergy between EC and

© 1996 Rapid Science Publishers monocytes in their production of IL-6 and GM–CSF, using a co-culture system.

### Materials and Methods

*Reagents:* PAF (1-*O*-alkyl-2-acetyl-*sn*-glycero-3-phosphocholine) was obtained from Bachem Fine Chemicals (Torrance, CA); TNF-α was purchased from Amersham and indomethacin was obtained from Sigma Chemical Co. (St Louis, MO).

*Isolation of endothelial cells:* EC were isolated from human umbilical vein as described previously.[39,40] Briefly, umbilical veins from individual cords were cannulated, washed with PBS, disodium EDTA ($5 \times 10^{-4}$ M; Sigma, St Louis, MO), and treated with 0.2% collagenase type V (Sigma) in PBS. After an incubation of 20 min at 37°C, detached cells were collected by washing twice with PBS, centrifuged and resuspended in complete medium consisting of Iscove medium (Flow, McLean, VA) supplemented with 10% heat-inactivated foetal bovine serum (FBS, Flow), penicillin (167 U/ml), streptomycin (200 μg/ml), amphotericin B (4 μg/ml), and 37.5 μg/ml EC growth supplement (CR–ECGS; Collaborative Research Inc., Lexington, MA). These primary culture cells were plated on 75-$cm^2$ tissue culture flasks (Falcon Oxnard, CA), fed three times a week and usually became confluent in 4–7 days. When cultures reached confluence, flasks were rinsed with disodium EDTA and then incubated for 2–3 min with EDTA containing 0.05% trypsin (Gibco, Grand Island, NY) to detach the cells. EC were washed, resuspended in complete medium and seeded in flasks precoated with 0.1% gelatin (Sigma) in Iscove medium.

EC were characterized by the detection of factor VIII-like antigen (von Willebrand's factor) by indirect immunofluorescence performed with a rabbit anti-vW factor antiserum (Calbiochem Behring, La Jolla, CA).

*Preparation of monocytes:* Peripheral blood was collected from healthy volunteers by venipuncture, diluted in PBS and underlaid with Ficoll–Hypaque[41] (Ficoll 400, Pharmacia, Uppsala, Sweden; Hypaque sodium, Winthrop Laboratories, Aurora, Ontario). After centrifugation for 30 min at 400 × ***g*** at room temperature, peripheral blood mononuclear leukocytes (PBML) were collected at the interface, washed twice in PBS and resuspended in Iscove medium containing 10% FBS and antibiotics. The cell suspension was composed of approximately 75–85% lymphocytes and 15–20% monocytes. Monocytes were separated by adherence to microexsudate-coated flasks[42] for 60 min at 37°C. After removal of nonadherent cells, containing 95–98% lymphocytes, the adherent cells were further incubated for 20 min at 37°C in the presence of monosodium EDTA and medium (1:1). This procedure yielded a cell population consisting of more than 95% pure monocytes as determined by non-specific esterase staining.

*Endothelial cells and monocyte co-cultures:* Confluent EC were detached from flasks, washed and resuspended in Iscove medium containing 5% FBS. Aliquots of $0.2 \times 10^6$ EC were preincubated for 24 h with either medium or graded concentrations of PAF. EC were then washed and incubated in the presence or absence of TNF-α (25 U/ml) and indomethacin (1 μM) for 4 h in Iscove medium supplemented with 0.25% BSA. Cells were washed again and incubated with $1 \times 10^6$ monocytes in Iscove medium containing 5% FBS. After a 24 h co-culture, cell-free supernatants were harvested and assayed for the presence of IL-6 and GM–CSF. In preliminary experiments, these conditions were found to be optimal for cytokine production.

*IL-6 and GM–CSF assay:* The activity of IL-6 was determined using IL-6-dependent cells of the murine hybridoma cell line B9 as described.[43,44] Briefly, serially diluted samples were dispensed into flat-bottomed 96-well microtitre plates (Limbro, McLean, MO). B9 cells, resuspended in RPMI 1640 medium + 5% FBS and $5 \times 10^{-5}$ M 2-mercaptoethanol, were added ($5 \times 10^3$ cells/well) and the plates were incubated at 37°C for 72 h. Proliferation of B9 cells was measured by a colorimetric assay using MTT (5 mg/ml; Sigma). Plates were then read at 595 nm using the BioRad Microplate Reader (BioRad, Richmond, CA). The activity of IL-6 in test supernatants was calculated on the basis of cellular growth obtained in the presence of various concentrations of a rIL-6 standard, using probit analysis of dilution curve data. Immunoreactive human GM–CSF was measured using the enzyme-linked immunosorbent assay (ELISA), commercially available from R & D Systems (Minneapolis, MN).

### Results

Unstimulated EC produced minimal quantities of IL-6, and their stimulation with TNF-α (25 U/ml) had a negligible effect (Fig. 1). In contrast, unstimulated monocytes produced significant amounts of IL-6. When these monocytes were co-cultured with resting EC, an additive effect was observed. In contrast, when they were co-cultured with TNF-treated EC, a synergistic three-

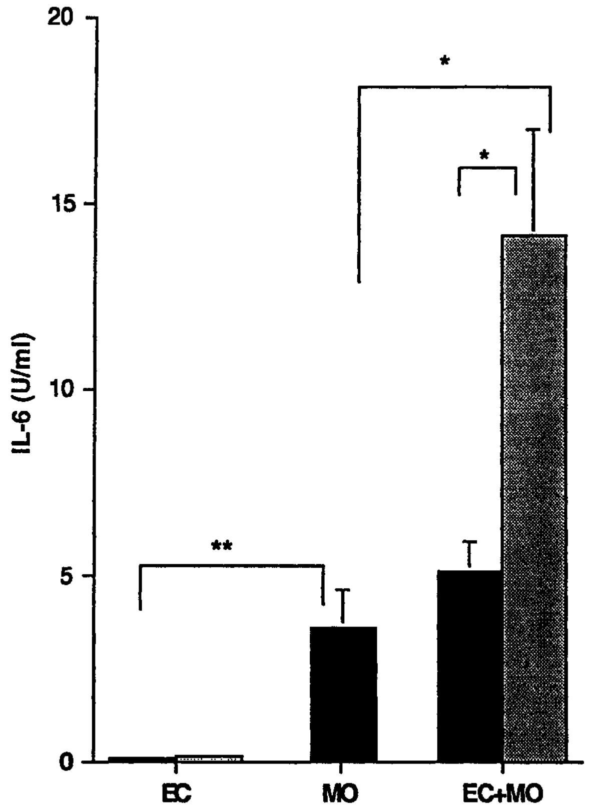

FIG. 1. Synthesis of IL-6 by endothelial cell (EC), monocytes (MO) or co-cultures of both EC and MO. EC were treated for 4 h with either medium or TNF-α (25 U/ml), washed and incubated for 24 h alone or with unstimulated MO. Cultures of monocytes alone were incubated for 24 h in medium. Cell-free supernatants were then collected and IL-6 activity was measured by the B9 cell proliferation assay. Data represent mean ± S.E.M. of seven to 14 experiments. Statistically significant effects were noted as: *$p < 0.005$; **$p < 0.0005$. Pretreatment of EC: ■, medium; ▩, PAF and TNF.

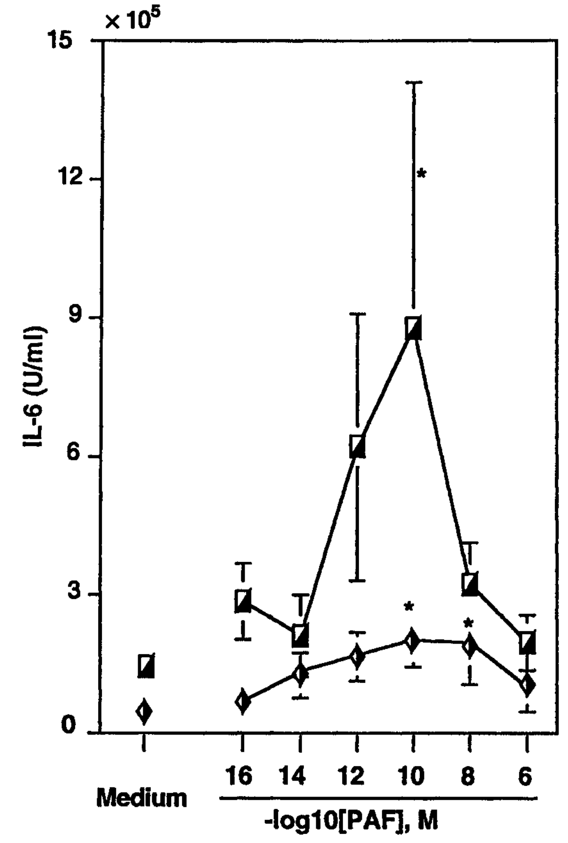

FIG. 2. Effect of PAF on IL-6 production by EC:MO co-cultures. EC were preincubated in the absence or presence of graded concentrations of PAF for 24 h, washed and exposed to medium or TNF-α (25 U/ml) for 4 h. After washing, EC were co-cultured with unstimulated monocytes for 24 h. Cell-free supernatants were then harvested for measurement for IL-6 activity. Data represent mean ± S.E.M. of four to 12 experiments. Statistically significant effects were noted as: *$p < 0.05$. ◪, with TNF; ◇, without TNF.

fold stimulation of IL-6 production was observed (Fig. 1).

Since PAF is involved in leukocyte–EC interactions, we examined whether PAF could modulate IL-6 production in EC–MO co-cultures. EC were pretreated with graded concentrations of PAF ($10^{-16}$–$10^{-6}$ M) before addition of monocytes. As shown in Fig. 2, PAF alone was at least as effective as TNF alone in stimulating IL-6 production in the co-cultures, with a maximal effect at $10^{-10}$ M PAF. When EC were pretreated with PAF and subsequently stimulated with TNF, their co-culture with monocytes resulted in a synergistic six-fold enhancement of IL-6 production.

In order to analyse the cellular requirements for the synergistic response to PAF and TNF, co-cultures were compared to EC or MO populations alone, as well as to co-cultures in which one of the cell populations was either killed by freeze–thawing (to preserve membrane structures) or fixed by paraformaldehyde treatment. As shown in Fig. 3, killed or fixed EC could no longer synergize with MO and IL-6 production reverted to that of MO alone. Similarly, killed or fixed MO could no longer synergize with EC and IL-6 levels fell to the minimal production observed with EC alone.

In contrast to IL-6, GM–CSF production was mainly observed in resting or stimulated EC, whereas monocytes alone produced negligible quantities of the cytokine (Fig. 4). Enhanced synthesis of GM–CSF was observed in co-cultures of untreated EC and MO but, whereas stimulated EC alone showed doubling of their GM–CSF production, addition of MO to stimulated EC had no further effect. The main cellular source of GM–CSF production was made evident in the co-cultures with one cell population killed by freeze–thawing or fixed with paraformaldehyde. Co-cultures of intact MO with fixed or killed EC showed little GM–CSF production, whereas co-cultures of intact EC with killed or fixed MO had responses similar to those of EC alone.

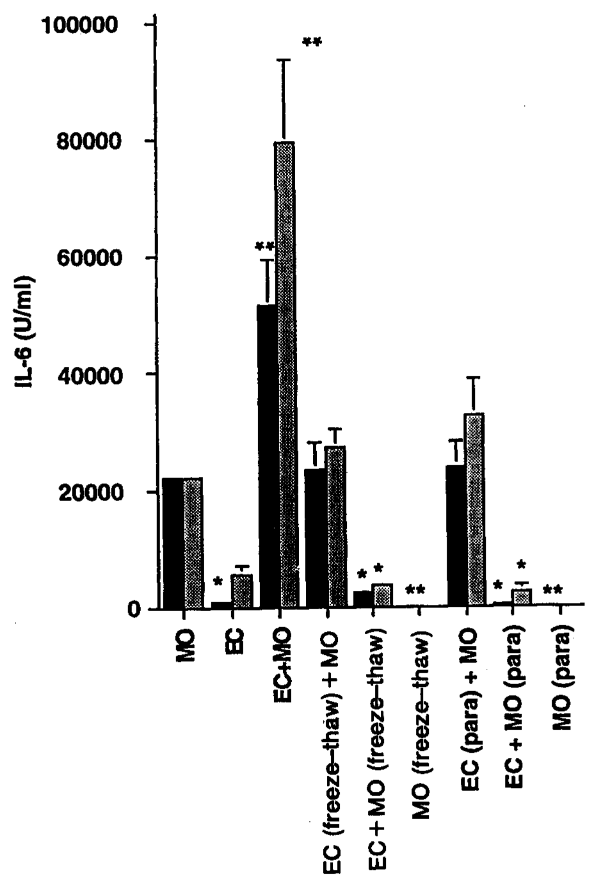

FIG. 3. Effect of prior lysis or fixation of either cell population on the production of IL-6 by EC:MO co-cultures. After treatment for 24 h with either medium or PAF ($10^{-10}$ M) + TNF-α (25 U/ml), EC were washed and either fixed with paraformaldehyde (0.05%) for 30 min, or lysed by three cycles of freezing (liquid nitrogen) and thawing (37°C). Thereafter EC were washed and incubated with monocytes. Alternatively, monocytes were fixed or lysed in the same manner and then washed and added to intact EC. After 24 h of co-culture, cell-free supernatants were harvested and assayed for IL-6 activity. Data represent mean ± S.E.M. of three experiments. Statistically significant effects were noted as: $^{*}p < 0.0001$; $^{**}p < 0.0005$, in comparison to monocytes alone. ■, Medium; ▩, TNF.

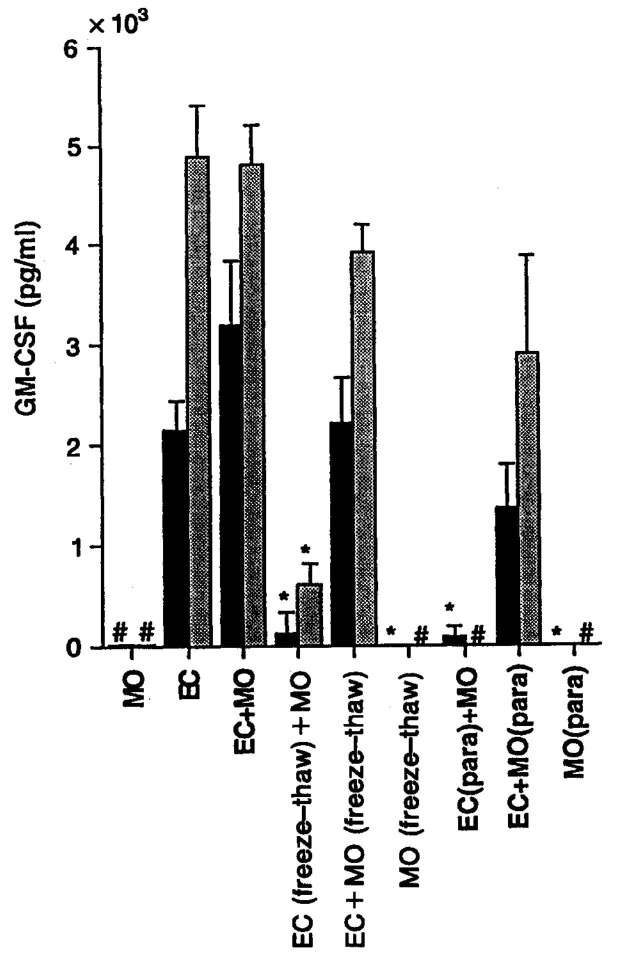

FIG. 4. Effect of prior lysis or fixation of either cell population on the production of GM-CSF by EC:MO co-cultures. After treatment for 24 h with either medium or PAF ($10^{-10}$ M) + TNF-α (25 U/ml), EC were washed and either fixed with paraformaldehyde (0.05%) for 30 min, or lysed by three cycles of freezing (liquid nitrogen) and thawing (37°C). Thereafter EC were washed and incubated with monocytes. Alternatively, monocytes were fixed or lysed in the same manner and then washed and added to intact EC. After 24 h of co-culture, cell-free supernatants were harvested and assayed for GM-CSF activity. Data represent mean ± S.E.M. of three experiments. Statistically significant effects were noted as: $^{*}p < 0.005$; $^{\#}p < 0.0005$, in comparison to EC alone. ■, Medium; ▩, PAF and TNF.

Because synergy in IL-6 production was dependent on live and fully functional MO and EC, and intact membranes did not substitute for live cells, we tested the requirement for actual cell–cell contact for the observed synergistic response. We used Costar transwell culture vessels in which MO were separated from EC by a polycarbonate, cell-impermeable membrane. As shown in Table 1, synergy in IL-6 production was maintained in spite of the physical separation of the two cell populations.

### Discussion

Our studies described here present evidence for contact-independent synergy in production of IL-6 by co-cultures of human MO and EC. Maximal effects were observed when resting MO were added to PAF-primed and TNF-treated EC, but a significant concentration-dependent enhancement of IL-6 production was also observed in co-cultures of MO and EC, when the latter were pretreated with PAF alone.

Since PAF can induce its own synthesis in EC[45] and since we have shown PAF to stimulate IL-6 production in EC, monocytes and alveolar macrophages,[46,47] it is possible that the observed synergy may be due, in part, to the action of *de novo* synthesized, EC-derived PAF and MO. This is unlikely, however, since EC retain most of the synthesized PAF in a cell-associated form and cell–cell contact was shown not to be necessary for the observed synergy. Moreover, EC mem-

**Table 1.** Contact-independent synergy of EC and MO in IL-6 production

| Cells | IL-6 (U/ml) |
|---|---|
| EC alone | |
| medium | 1200 ± 50 |
| PAF + TNF | 3020 ± 400 |
| MO alone | 16200 ± 4800 |
| MO + EC | |
| medium | 43600 ± 6440 |
| PAF + TNF | 62100 ± 5200 ($p < 0.05$) |
| MO + EC/transwells | |
| medium | 40400 ± 2200 |
| PAF + TNF | 52800 ± 1100 ($p < 0.02$) |

EC were preincubated with medium or PAF ($10^{-10}$ M) + TNF-α (25 U/ml) as indicated in Materials and Methods and thereafter either cultured alone or with unstimulated MO. Alternatively, EC were cultured in Costar transwells, separated from MO by a cell-impermeable polycarbonate membrane. After 24 h of culture, cell-free supernatants were harvested for assays of IL-6 content. Supernatants from either side of the transwell membrane had equivalent contents of IL-6.

branes alone were not sufficient to trigger MO synthesis of IL-6, as demonstrated by the freeze–thaw experiments: MO cultured with lysed, but otherwise intact PAF-primed EC produced no more IL-6 than MO alone.

In contrast to IL-6, GM–CSF production was mainly derived from EC and their stimulation with PAF and TNF resulted in enhanced production of the cytokine. Since GM–CSF can increase IL-6 production by monocytes,[23] it remains to be tested whether GM–CSF plays a role in the synergy in IL-6 production observed in our co-cultures. The transwell experiments suggest that a soluble factor from either of the two cell types, or from both cell types, is essential for the up-regulated production of cytokines. Further studies will help determine the nature of such factor(s).

In summary, exposure to EC and the inflammatory stimuli PAF and TNF resulted not only in their enhanced production of GM–CSF, but also in their ability to synergize with MO in augmented IL-6 production. Interestingly, this synergy did not require cell–cell contact between EC and MO, in contrast to many other synergistic events which depend on cell contact and involve various combinations of cell adhesion molecules.

### References

1. Harlan JM. Leukocyte–endothelial interactions. *Blood* 1985; **65:** 513–525.
2. Collins T, Korman AJ, Wake CT, *et al.* Immune interferon activates multiple class II major histocompatibility complex genes and the associated invariant gene in human endothelial cells and dermal fibroblasts. *Proc Natl Acad Sci USA* 1984; **81:** 4917–4921.
3. Collins T, Lapierre LA, Fiers W, Strominger JL, Pober JS. Recombinant human tumor necrosis factor increases mRNA levels and surface expression of HLA-A,B antigens in vascular endothelial cells and dermal fibroblasts *in vitro*. *Proc Natl Acad Sci USA* 1986; **83:** 446–450.
4. Bevilacqua MP, Schleef RR, Gimbrone Jr MA, Loskutoff DJ. Regulation of the fibrinolytic system of cultured human vascular endothelium by interleukin 1. *J Clin Invest* 1986; **78:** 587–591.
5. Camussi G, Aglietta M, Coda R, Bussolino F, Piacibello W, Tetta C. Release of platelet-activating factor (PAF) and histamine. II. The cellular origin of human PAF: monocytes, polymorphonuclear neutrophils and basophils. *Immunology* 1981; **42:** 191–199.
6. Camussi G, Aglietta M, Malavasi F, Tetta C, Piacibello W, Sanavio F, Bussolino F. The release of platelet-activating factor from human endothelial cells in culture. *J Immunol* 1983; **131:** 2397–2402.
7. Camussi G, Bussolino F, Tetta C, Piacibello W, Aglietta M. Biosynthesis and release of platelet-activating factor from human monocytes. *Int Arch Allergy Appl Immunol* 1983; **70:** 245–251.
8. Whatley RE, Zimmerman GA, McIntyre TM, Taylor R, Prescott SM. Production of platelet-activating factor by endothelial cells. *Semin Thromb Hemost* 1987; **13**(4): 445–453.
9. Benveniste J, Henson PM, Cochrane CG. Leukocyte-dependent histamine release from rabbit platelets: the role of IgE, basophils and platelet-activating factor. *J Exp Med* 1972; **136:** 1356–1377.
10. Demopoulos CA, Pinckard RN, Hanahan DJ. Platelet-activating factor. Evidence for 1-O-alkyl-2-acetyl-*sn*-glycero-3-phosphorylcholine as the active component (a new class of lipid chemical mediators). *J Biol Chem* 1979; **254:** 9355–9358.
11. Prescott S, Zimmerman GA, McIntyre TM. Platelet-activating factor. *J Biol Chem* 1990; **265:** 17381–17384.
12. Rola-Pleszczynski M, Pignol B, Pouliot C, Braquet P. Inhibition of human lymphocyte proliferation and interleukin 2 production by platelet activating factor (PAF-acether): reversal by a specific antagonist, BN 52021. *Biochem Biophys Res Commun* 1987; **142:** 754–760.
13. Rola-Pleszczynski M, Pouliot C, Turcotte S, Pignol B, Braquet P, Bouvrette L. Immune regulation by platelet-activating factor. I. Induction of suppressor cell activity in human monocytes and CD8+ T cells and of helper cell activity in CD4+ T cells. *J Immunol* 1988; **140:** 3547–3552.
14. Poubelle P, Gingras D, Demers C, Dubois C, Harbour D, Rola-Pleszczynski M. Platelet activating factor (PAF-acether) enhances the concomitant production of tumor necrosis factor alpha and interleukin 1 by subsets of human monocytes. *Immunology* 1991; **72:** 181–187.
15. Thivierge M, Rola-Pleszczynski M. Platelet-activating factor (PAF) enhances interleukin-6 production by alveolar macrophages. *J Aller Clin Immunol* 1992; **90:** 796–802.
16. Bussolino F, Camussi G, Aglietta M, *et al.* Human endothelial cells are targets for platelet-activating factor. I. Platelet-activating factor induces changes in cytoskeleton structures. *J Immunol* 1987; **139:** 2439–2446.
17. Braquet P, Bourgain R, Mencia-Huerta JM. Effect of platelet-activating factor on platelets and vascular endothelium. *Sem Thromb Hemos* 1989; **15:** 184–196.
18. Zimmerman GA, McIntyre TM, Mehra M, Prescott SM. Endothelial cell-associated platelet-activating factor: a novel mechanism for signalling intercellular adhesion. *J Cell Biol* 1990; **110:** 529–540.
19. Holzinger C, Weissinger E, Zuckermann A, *et al.* Effects of interleukin-1, -2, -4, -6, interferon-gamma and granulocyte/macrophage colony stimulating factor on human vascular endothelial cells. *Immunol Lett* 1993; **35:** 109–118.
20. Jirik FR, Podor TJ, Hirano T, Kishimoto T, Loskutoff DJ, Carson DA, Lotz M. Bacterial lipopolysaccharide and inflammatory mediators augment IL-6 secretion by human endothelial cells. *J Immunol* 1989; **142:** 144–147.
21. Loppnow H, Libby P. Adult human vascular endothelial cells express the IL-6 gene differentially in response to LPS or IL-1. *Cell Immunol* 1989; **122:** 493–503.
22. Sironi M, Breviario F, Proserpio P, *et al.* IL-1 stimulates IL-6 production in endothelial cells. *J Immunol* 1989; **142:** 549–553.
23. Bauer J, Ganter U, Geiger T, *et al.* Regulation of interleukin-6 expression in cultured human blood monocytes and monocyte-derived macrophages. *Blood* 1988; **72:** 1134–1140.
24. Tosato G, Seamon KB, Goldman ND, *et al.* Monocyte-derived human B cell growth factor identified as interferon-β2 (BSF-2, IL-6). *Science* 1988; **239:** 502–504.
25. Navarro S, Debili N, Bernaudin JF, Vainchenker W, Doly J. Regulation of the expression of IL-6 in human monocytes. *J Immunol* 1989; **142:** 4339–4345.
26. Content J, De Wit L, Pierard D, Derynck R, De Clercq E, Fiers W. Secretory proteins induced in human fibroblasts under conditions used for the production of interferon β. *Proc Natl Acad Sci USA* 1982; **79:** 2768–2772.
27. Bagby Jr GC, Dinarello CA, Wallace P, Wagner C, Hefeneider S, McCall E. Interleukin 1 stimulates granulocyte macrophage colony-stimulating activity release by vascular endothelial cells. *J Clin Invest* 1986; **78:** 1316–1323.
28. Broudy VC, Kaushansky K, Harlan JM, Adamson JW. Interleukin 1 stimulates human endothelial cells to produce granulocyte-macrophage colony-simulating factor. *J Immunol* 1987; **139:** 464–468.
29. Segal GM, McCall E, Stueve T, Bagby Jr GC. Interleukin 1 stimulates endothelial cells to release multilineage human colony-stimulating activity. *J Immunol* 1987; **138:** 1772–1778.
30. Sieff CA, Tsai S, Faller DV. Interleukin 1 induces cultured human endothelial cell production of granulocyte-macrophage colony-stimulating factor. *J Clin Invest* 1987; **79:** 48–51.
31. Fibbe WE, Daha MR, Hiemstra PS, *et al.* Interleukin 1 and poly (rI).poly (rC) induce production of granulocyte CSF, macrophage CSF, and granulocyte-macrophage CSF by human endothelial cells. *Exp Hematol* 1989; **17:** 229–234.
32. Broudy VC, Kaushansky K, Segal GM, Harlan JM, Adamson JW. Tumor necrosis factor type α stimulates human endothelial cells to produce granulocyte/macrophage colony-stimulating factor. *Proc Natl Acad Sci USA* 1986; **83:** 7467–7471.
33. Munker R, Gasson J, Ogawa M, Koeffler HP. Recombinant human TNF induces production of granulocyte-monocyte colony-stimulating factor. *Nature* 1986; **323:** 79–82.
34. Cline MJ, Golde DW. Production of colony-stimulating activity by human lymphocytes. *Nature* 1974; **248:** 703–704.
35. Wodnar-Filipowicz A, Heusser CH, Moroni C. Production of the haemopoietic growth factors GMCSF and interleukin-3 by mast cells in response to IgE receptor-mediated activation. *Nature* 1989; **339:** 150–152.
36. Thorens B, Mermod JJ, Vassalli P. Phagocytosis and inflammatory stimuli induce GM–CSF mRNA in macrophages through posttranscriptional regulation. *Cell* 1987; **48:** 671–679.
37. Gamble JR, Elliott MJ, Jaipargas E, Lopez AF, Vadas MA. Regulation of human monocyte adherence by granulocyte-macrophage colony-stimulating factor. *Proc Natl Acad Sci USA* 1989; **86:** 7169–7173.
38. Smith PD, Lamerson CL, Wong HL, Wahl LM, Wahl SM. Granulocyte-macrophage colony-stimulating factor stimulates human monocyte accessory cell function. *J Immunol* 1990; **144:** 3829–3834.
39. Jaffe EA, Nachman RL, Becker CG, Minick CR. Culture of human endothelial cells derived from umbilical veins. Identification by morphologic and immunologic criteria. *J Clin Invest* 1973; **52:** 2745–2756.
40. Moraes JR, Stastny P. A new antigen system expressed in human endothelial cells. *J Clin Invest* 1977; **60:** 449–454.
41. Böyum A. Isolation of mononuclear cells and granulocytes from human blood. *Scand J Clin Lab Invest* 1968; **21**(suppl. 97): 77–89.
42. Douglas SD, Zuckerman SM, Ackerman SK. Obtaining and culturing human monocytes. In: Adams DO, Edelson PS, Koren M, eds. *Methods for Studying Mononuclear Phagocytes.* New York: Academic Press, 1981; 33.
43. Lansdorp PM, Aarden LA, Calafat J, Zeiljemaker WP. A growth-factor dependent B-cell hybridoma. *Curr Top Microbiol Immunol* 1986; **132:** 105–113.
44. Aarden LA, DeGroot ER, Schaap OL, Lansdorp PM. Production of hybridomas growth factor by human monocytes. *Eur J Immunol* 1987; **17:** 1411–1416.
45. Heller R, Bussolino F, Ghigo D, Garbarino G, Pescarmona G, Till U, Bosia A. Human endothelial cells are targets for platelet-activating factor. II. Platelet-activating factor induces platelet-activating factor synthesis in human umbilical vein endothelial cells. *J Immunol* 1992; **149:** 3682–3688.
46. Rola-Pleszczynski M, Bouvrette L, Thivierge M, Lacasse C. Platelet-activating factor enhances interleukin 6 production by monocytes, alveolar macrophages and endothelial cells. *FASEB J* 1990; **4:** A1713.
47. Rola-Pleszczynski M, Thivierge M, Gagnon N, Lacasse C, Stankova J. Differential regulation of cytokine and cytokine receptor genes by PAF, $LTB_4$ and $PGE_2$. *J Lipid Mediat* 1993; **6:** 175–181.

ACKNOWLEDGEMENTS. This work was supported by a grant from the Medical Research Council of Canada and by a Studentship to C.L. from the Heart and Stroke Foundation of Canada.

**Received 19 October 1995; accepted in revised form 8 December 1995**
